# A Nitrogen Molecular Sensing System, Comprised of the *ALLANTOINASE* and *UREIDE PERMEASE 1* Genes, Can Be Used to Monitor N Status in Rice

**DOI:** 10.3389/fpls.2018.00444

**Published:** 2018-04-18

**Authors:** Dong-Keun Lee, Mark C. F. R. Redillas, Harin Jung, Seowon Choi, Youn Shic Kim, Ju-Kon Kim

**Affiliations:** Graduate School of International Agricultural Technology and Crop Biotechnology Institute, GreenBio Science and Technology, Seoul National University, Pyeongchang, South Korea

**Keywords:** allantoin, luciferase, molecular sensor, nitrogen, rice, ureide

## Abstract

Nitrogen (N) is an essential nutrient for plant growth and development, but its concentration in the soil is often insufficient for optimal crop production. Consequently, improving N utilization in crops is considered as a major target in agricultural biotechnology. However, much remains to be learnt about crop N metabolism for application. In this study, we have developed a molecular sensor system to monitor the N status in rice (*Oryza sativa*). We first examined the role of the ureide, allantoin, which is catabolized into allantoin-derived metabolites and used as an N source under low N conditions. The expression levels of two genes involved in ureide metabolism, *ALLANTOINASE* (*OsALN*) and *UREIDE PERMEASE 1* (*OsUPS1*), were highly responsive to the N status. *OsALN* was rapidly up-regulated under low N conditions, whereas *OsUPS1* was up-regulated under high N conditions. Taking advantage of the responses of these two genes to N status, we generated transgenic rice plants harboring the molecular N sensors, *proALN::ALN-LUC2* and *proUPS1::UPS1-LUC2*, comprising the gene promoters driving expression of the luciferase reporter. We observed that expression of the transgenes mimicked transcriptional regulation of the endogenous *OsALN* and *OsUPS1* genes in response to exogenous N status. Importantly, the molecular N sensors showed similar levels of specificity to nitrate and ammonium, from which we infer their sensing abilities. Transgenic rice plants expressing the *proUPS1::UPS1-LUC2* sensor showed strong luminescence under high exogenous N conditions (>1 mM), whereas transgenic plants expressing the *proALN::ALN-LUC2* sensor showed strong luminescence under low exogenous N conditions (<0.1 mM). High exogenous N (>1 mM) substantially increased internal ammonium and nitrate levels, whereas low exogenous N (<0.1 mM) had no effect on internal ammonium and nitrate levels, indicating the luminescence signals of molecular sensors reflect internal N status in rice. Thus, *proALN::ALN-LUC2* and *proUPS1::UPS1-LUC2* represent N molecular sensors that operate over a physiological and developmental range in rice.

## Introduction

Nitrogen (N), an integral component of proteins, nucleic acids and other biomolecules, is a key element required for plant growth and development (Castro-Rodriguez et al., 2017). Crops typically have an insufficient natural supply of N in the soil and so N fertilizers are applied to enhance yield. However, the use, and often over-use, of N fertilizer have undesirable effects on the environment and the agricultural economy. To address this issue, an important goal of crop development and management is to reduce N fertilizer usage while maintaining yield. On average, crops use only 25–50% of the applied N fertilizer (Undurraga et al., 2017), which means that improving crop only by management and breeding approaches is challenging. An alternative is to develop biotechnological strategies to improve crop N utilization, but at present the molecular basis of N metabolism is not well understood, which represents a major barrier to crop improvement.

N metabolism can be divided into phases of uptake, assimilation and mobilization. Plant roots absorb N in several forms, such as nitrate, ammonium, and other organic molecules. Among them, nitrate and ammonium are the major forms in aerobic and flooded environments, respectively (Oldroyd and Dixon, 2014), and their uptake is controlled by low- and high-affinity transporters. High-affinity transporters are induced to acquire under low N conditions, whereas low-affinity transporters absorb N sources under high N conditions (Li et al., 2017). Following the first identification of a nitrate transporter, *CLH1*/*AtNRT1.1* in *Arabidopsis thaliana* (Tsay et al., 1993), five more *A. thaliana* (*AtNRT1.2, AtNRT2.1, AtNRT2.2, AtNRT2.4*, and *AtNRT2.5*) and six rice (*Oryza sativa)* (*OsNRT1, OsNRT2.1, OsNRT2.2, OsNRT2.4, OsNRT1.1B*, and *OsNPF2.4*) nitrate transporters were shown to be involved in nitrate uptake from the external environment (Huang et al., 1999; Lin et al., 2000; Li et al., 2007; Feng et al., 2011; Yan et al., 2011; Kiba et al., 2012; Lezhneva et al., 2014; Hu et al., 2015; Xia et al., 2015). In other studies, *AtAMT1;1, AtAMT1;3*, and *AtAMT1;5* were identified in *A. thaliana* as transporters involved in ammonium uptake from the external environment (Gazzarrini et al., 1999; Yuan et al., 2007), while *OsAMT1;1* and *OsAMT3;1* were functionally characterized as ammonium uptake transporters from rice (Hoque et al., 2006; Bao et al., 2015; Li et al., 2016). Nitrate and ammonium uptake are considered to be important targets for improving crop N metabolism, and ammonium is recognized as a key N source in rice growing under anaerobic conditions; however, only limited information is available about N uptake mechanisms in rice.

After nitrate is absorbed by roots, it is sequentially reduced by nitrate reductase to nitrite and then by nitrite reductase to ammonium (Cheng et al., 1991; Xu et al., 2012). Plants also assimilate inorganic ammonium to glutamine via the GS (glutamine synthetase)-GOGAT (glutamate synthase) cycle (Thomsen et al., 2014). Assimilated amino acids are then mobilized to the shoots via the xylem. For example, the ammonium taken up by rice roots is almost all assimilated into glutamine, with a small amount to asparagine, both of which are common N mobile compounds (Yoneyama, 1986; Yoneyama et al., 2016). Finally, N compounds in the shoots are exported to their sites of usage via phloem loading (Tegeder, 2014). Much remains to be learnt about the mechanism that controls the root-to-shoot transport of amino acids. However, in addition to N-containing amino acids, nitrate and ammonium themselves also undergo root-to-shoot transport via xylem loading. Nitrate transporters, including AtNRT1.5, AtNRT1.8, AtNRT1.9, AtNPF2.3, OsNRT1.1B, OsNPF2.2, OsNRT2.3a, and OsNPF2.4, have been identified in *A. thaliana* and rice, and shown to be important in root-to-shoot nitrate transport (Lin et al., 2008; Li et al., 2010, 2015; Wang and Tsay, 2011; Tang et al., 2012; Hu et al., 2015; Taochy et al., 2015; Xia et al., 2015). Recently, Fang et al. (2017) demonstrated that expression of *OsNPF7.3* is induced by organic Ns, contributing to N allocation for improving gain yield in rice. However, the molecular mechanism responsible for xylem loading of ammonium remains unknown in both species.

In legumes, ureides comprise up to 90% of the total N source (Herridge et al., 1978) and are the main N compounds involved in long-distance transport (Todd et al., 2006; Tegeder, 2014). The dominant forms of ureides, allantoin and allantoic acid, which are also intermediates of a purine catabolic pathway, are synthesized in the root and transported to the shoot, where they are catabolized to generate ammonium for re-assimilation to amino acids (Smith and Atkins, 2002). The catabolic processes are regulated by enzymes including allantoinase (ALN). Ureide permease (UPS), which is localized in the plasma membrane, is known to be a ureide transporter, and may also be involved in the root-to-shoot transport of ureides (Desimone et al., 2002; Pelissier et al., 2004). It was reported that RNAi-mediated repression of the soybean (*Glycine max*) *UPS* genes, *GmUPS1* and *GmUPS1*, resulted in decreased export of allantoin and allantoic acid from nodules, giving rise to N-deficiency phenotypes in the shoots (Collier and Tegeder, 2012). Conversely, overexpression of common bean (*Phaseolus vulgaris*) *PvUPS1* in soybean increased the export of allantoin and allantoic acid from the nodules, resulting in an improved shoot N supply (Carter and Tegeder, 2016). These results are consistent with a role for *UPS* genes in the translocation of ureide between organs. Non-legumes also have ureide biosynthetic and catabolic genes, and *A. thaliana* can utilize allantoin as an N source when it represents the only N source (Desimone et al., 2002; Yang and Han, 2004).

The development of crops with improved N metabolism has been slow, in part due to the absence of a molecular N sensor system, which is a monitoring tool to easily detect N status in plants. Fluorescent-based biosensors for N sources such as AmTrac for ammonium and NiTrac for nitrate were developed to study transporter activity *in vivo* (Michele et al., 2013; Ho and Frommer, 2014). Both biosensors sensitively show changes in concentration-dependent fluorescence intensity in response to each specific N source in yeast. However, there is no report on a molecular N sensor system to directly detect internal N status in plants. In this study, we describe the development of an N molecular sensor system, based on *OsALN* and *OsUPS1* genes as an approach to monitor internal N status in rice plants. First, we characterized the role of allantoin as an N source in rice plants, and we then used this information to develop *in vivo* molecular N sensors in transgenic rice plants.

## Materials and Methods

### Growth Conditions and Chlorophyll Measurements

To investigate the potential role of allantoin as an N source in rice, the Dongjin rice cultivar (*O. sativa Japonica*) was grown for 9 days in a growth chamber (16 h light/8 h dark at 28–30°C). Seeds were sterilized with 70% ethanol for 20 min followed by 50% chloral hydrate for 30 min and were placed on solid growth media (GM-N) (pH 5.8), consisting of 0.44% MS (Murashige and Skoog) salts without a supplemental N source (Caisson Labs, United States), 3% sucrose and 0.5% agar (Sigma, United States). We set 11 ∼ 12 rice plants on a 9 cm (diameter)/2 cm (height) circle petri dish including ∼70 ml growth media. As sole N source, various concentrations of allantoin (0, 0.025, 0.25, 2.5 or 25 mM final concentration, Sigma) were added to the GM-N. Positive controls were Dongjin plants grown on solid growth media (GM+N), which included MS salts with N sources (20 mM ammonium nitrate and 19 mM potassium nitrate) (Caisson Labs, United States), 3% sucrose and 0.5% agar. The height of 9-day-old rice plants (*n* = 40 for each allantoin concentration) were measured. Additionally, since chlorophyll content is closely correlated with N status (Xiong et al., 2015; Yuan et al., 2016), SPAD values were determined at a distance 2/3 from the leaf base to the apex of the second uppermost leaf of 16-day-old plants (*n* = 40 for each allantoin concentration) using a SPAD 502 chlorophyll meter (Konica Minolta, Japan).

### N Starvation and Re-application Experiment

Rice seeds (Dongjin) were germinated and grown in soil for 2 weeks, then, seedlings were washed to remove soil, transferred to pots containing water and grown for 2 more days. Seedlings were transferred to and grown for 5 days in Yoshida solution, including 2.8 mM NH_4_NO_3_ as the N source (Yoshida et al., 1976). Twenty one days after germination, seedlings were grown under N starvation conditions (Yoshida solution without NH_4_NO_3_) for 10 more days. After N starvation, NH_4_NO_3_ was re-introduced into the solution (2.8 mM final concentration), which corresponded to the N re-application condition. Sampling time points are indicated in **Figure 2A**. Tissues to be used for qRT-PCR and allantoin metabolite analysis were frozen and powdered in liquid nitrogen immediately after sampling.

### Allantoin Metabolite Analysis

Allantoin quantification was performed by differential analyses of glyoxylate derivatives (Vogels and Van der Drift, 1970). Briefly, the analysis was divided into four assays: glyoxylate (assay A), ureidoglycolate (assay B), allantoate (assay C), and allantoin (assay D). Tissue powders were mixed with ice-cold deionized water, held at 4°C for 1 h and then centrifuged at 13,000 *g* for 30 min at 4°C. The supernatant was filtered through a 0.20 μm cellulose acetate filter (Advantec, JAPAN) to produce the plant extract. For assay A, 100 μl plant extract was diluted to 500 μl with water and 100 μl each of 0.4 M KH_2_PO_4_ and 0.33% phenylhydrazine-HCl was added. The mixture was placed in an ice-cold water bath and mixed with 500 μl of cold (4°C) concentrated HCl. After adding 100 μl of 1.65% potassium ferricyanide, the sample was left for 15 min at room temperature (RT) and the absorbance of the solution at 535 nm measured using a spectrophotometer (Tecan Infinite M200 NanoQuant, Switzerland). For assay B, 100 μl of 0.5 M NaOH was added to 100 μl plant extract and the sample diluted to 500 μl with water. The mixture was allowed to stand at RT for 2 min followed by the same procedure as for assay A. For assay C, 100 μl of 0.15 M HCl was added to 100 μl plant extract and the sample diluted to 500 μl with water. The mixture was then boiled in a water bath for 5 min before cooling to RT, followed by the assay A procedure. For assay D, 100 μl of 0.5 M NaOH was added to 100 μl plant extract and the sample diluted to 400 μl with water. The mixture was boiled in a water bath for 10 min, cooled to RT, 100 μl of 0.65 M HCl was added and the sample boiled again in a water bath for 5 min followed by the assay A procedure. The allantoin concentration was calculated by subtracting the value of assay C from the value of assay D. The concentration of allantoic acid was determined by subtracting the value of assay B from the value of assay C. The ureidoglycolate concentration was determined by subtracting the value of assay A from the value of assay B. Two biological and two technical replicates were analyzed for all quantitative experiments.

### Quantitative Real Time (qRT) PCR Analysis

Total RNA was extracted from leaves and roots of rice plants grown under N starvation and re-application conditions using a Qiagen RNeasy plant mini kit (Qiagen, United States). One microgram of total RNA was used to synthesize cDNA with an oligo dT primer using RevertAid reverse transcriptase (Thermo Fisher Scientific, United States). Real-time PCR analysis was performed using the 2X Real-Time PCR Smart mix with Evagreen (Solgent, South Korea) and the Mx3000P Real-Time PCR system (Agilent Technologies, United States). The *OsUBI1* (*UBIQUITIN 1*, Os06g0681400) gene was used as an internal control for expression normalization. Two biological and two technical replicates were analyzed for all quantitative experiments. Gene specific primers used for qRT-PCR are listed in Supplementary Table S1.

### Sensor Constructs and Rice Transformation

A *LUCIFERASE 2* (*LUC2*) DNA fragment was amplified by PCR from the pGL4.10 vector (Promega, United States) using the PrimeSTAR HS DNA Polymerase (Takara, Japan) and primers (ATCTCGAGATGGAAGATGCCAAAAACATTAAGAAGGG and ATGGATCCTATCACGGCGATCTTGCCGCCC), and ligated into the rice pPZP-BAR transformation vector (Hajdukiewicz et al., 1994). DNA fragments corresponding to 2 and 2.2 kb of the *OsALN* and *OsUPS1* promoters, respectively, were amplified from rice cultivar Nipponbare genomic DNA using the PrimeSTAR HS DNA Polymerase. The primers used were AGCTCAAGCTAAGCTTCGTCCACTCGCCGGAGAACAT and CATCTTCCATCTCGAGTCGCGTGCGCGTGGG for the *OsALN* promoter and AGCTCAAGCTAAGCTTATTCGGTTGCATTGCGGTGCTGC and CAAGATACATAAGCTTGATCTCGCCGGAGCCGGAGAAGA for the *OsUPS1* promoter. DNA fragments corresponding to the coding regions of *OsALN* and *OsUPS1* were amplified using the PrimeSTAR HS DNA Polymerase and Reverse Transcription System (Promega, United States), from total RNA extracted from whole 2-week-old Nipponbare plants. The primers used were GCGCACGCGACTCGAGATGGCGATGGCGGCGG and CATCTTCCATCTCGAGTTTGGCGAGGATTGGAGCACC for the *OsALN* cDNA and AGCTCAAGCTAAGCTTATGTATCTTG TGAAGGATATCGGCGG and CATCTTCCATCTCGAGGAGCGGCCTCCGGTGCGC for the *OsUPS1* cDNA. The promoters and cDNAs were ligated into the pPZP-BAR vector using the In-Fusion HD Cloning kit (Takara, Japan) to finally make the *proALN::ALN-LUC2* and *proUPS1::UPS1-LUC2* constructs (Supplementary Figures S3A,B). Transgenic plants (Dongjin) were generated by *Agrobacterium tumefaciens* (LBA4404)-mediated co-cultivation, as previously described (Jang et al., 1999). To determine the transgene copy number in the T_0_ generation (*n* = 70 for *proALN::ALN-LUC2* and *n* = 66 for *proUPS1::UPS1-LUC2*), TaqMan q-PCR was performed as previously described (Lee et al., 2016). Five independent homozygous lines with a single copy of the transgene from the T_2_ lines were used for subsequent studies.

### Luciferase Assay

Two growth conditions were used to examine luminescence in the transgenic rice plants: long term incubation under a specific N condition, and short term incubation under high N conditions. For long term incubation under a specific N condition, seeds from T_3_ homozygous *proALN::ALN-LUC2* or *proUPS1::UPS1-LUC2* lines were placed on GM+N or GM-N medium. The plates were incubated vertically in the dark for 2 days and transferred to light (16 h light/8 h dark cycle) at 28°C for 3 days. To determine the luciferase activity, 1 mM luciferin (Gold Biotechnology, United States) in a 0.01% Triton X-100 solution was sprayed onto the transgenic plants and luminescence was measured using an IVIS Lumina 3 system and Living Image 4.5.2 software (PerkinElmer, United States). For short term incubation under high N conditions, seeds from T_3_ homozygous *proALN::ALN-LUC2* or *proUPS1::UPS1-LUC2* lines were placed on GM-N medium for 4 days. Ammonium nitrate (100 mM; Sigma, United States) was added to the transgenic plants, which were then incubated for 1 day under light conditions. One mM luciferin in a 0.01% Triton X-100 solution was sprayed onto the transgenic plants and luminescence was measured as above. Luminescence intensity (average radiance [p/s/cm^2^/sr]) of the transgenic plants grown under both long and short term incubation was measured using the same size of subject ROI (region of interest) in each rice shoot. We used five independent T_3_ lines for each *in vivo* molecular sensor, which were homozygotes with a single copy of the transgene (*n* = 10 plants for each line). Values are the means ± SD (standard deviation) of five independent lines for each molecular N sensor (total *n* = 50 for each sensor). Non-transgenic Dongjin plants (NT) were used as a control for normalization.

### Determination of N Substrate Specificity and Sensitivity of N Sensors

To determine the N substrate specificity and sensitive range of each N sensor, 3 T_3_ homozygous lines for sensor were germinated on solid GM-N medium (pH 5.8) and vertically incubated in the dark for 1 days and transferred to light (16 h light/8 h dark cycle) at 28°C for 3 days. We set 9 rice plants on a 12.5 cm by 12.5 cm/2 cm (height) square petri dish including ∼100 ml growth media. The 4-day-old rice plants (*n* = 9 plants per a concentration for each line) were treated with 1.0 M, 100 mM, 10 mM, 1 mM, 100 μM, 10 μM, 1 μM, or 0.1 μM ammonium nitrate, ammonium sulfate or potassium nitrate, and incubated for 1 day in a growth chamber under 16 h light/8 h dark cycle at 28°C. After spraying 1 mM luciferin in a 0.01% Triton X-100 solution onto the plants, luminescence (average radiance [p/s/cm^2^/sr]) was measured as above. The controls for normalization used were *proUPS1::UPS1-LUC2* plants grown on GM-N for *proUPS1::UPS1-LUC2* lines or *proALN::ALN-LUC2* plants grown on GM-N with 1 M N substrate for the *proALN::ALN-LUC2* lines.

### Determination of Internal Ammonium and Nitrate Contents

Wild type Dongjin rice seeds were germinated on solid GM-N medium (pH 5.8) and vertically incubated in the dark for 1 days and transferred to light (16 h light/8 h dark cycle) at 28°C for 3 days. We set 9 rice plants on a 12.5 cm by 12.5 cm/2 cm (height) square petri dish including ∼100 ml growth media. The 4-day-old rice plants were treated with 100 mM, 10 mM, 1 mM, 100 μM, 10 μM, or 1 μM ammonium nitrate, and incubated for 1 day in a growth chamber under 16 h light/8 h dark cycle at 28°C. For analysis of ammonium content, the Berthelot method was followed with some modification. 0.1 g (fresh weight) rice shoots were ground and mixed with 500 μl of 2% sulfosalicylic acid (Sigma). After incubation for 2 min at room temperature and centrifuging briefly, 10 μl of supernatant was reacted with 50 μl of Reagent A (0.33 M sodium phenolate in 2 N NaOH with pH adjusted to pH 13), 50 μl of Reagent B (0.01% sodium nitroprussiate) and 50 μl of Reagent C (26 mM sodium hypochlorite). After 1 h incubation at room temperature, ammonium level was measured by a spectrophotometer (NanoQuant, Infinite M200, Switzerland) at 635 nm wavelength. Results were then compared through a standard curve using ammonium sulfate (Sigma) as standard compound. Analysis of nitrate content was performed with 0.1 g (fresh weight) rice shoots as described by Xu et al. (2016).

## Results

### Allantoin Can Provide a Source of N in Rice Plants

**FIGURE 1:**
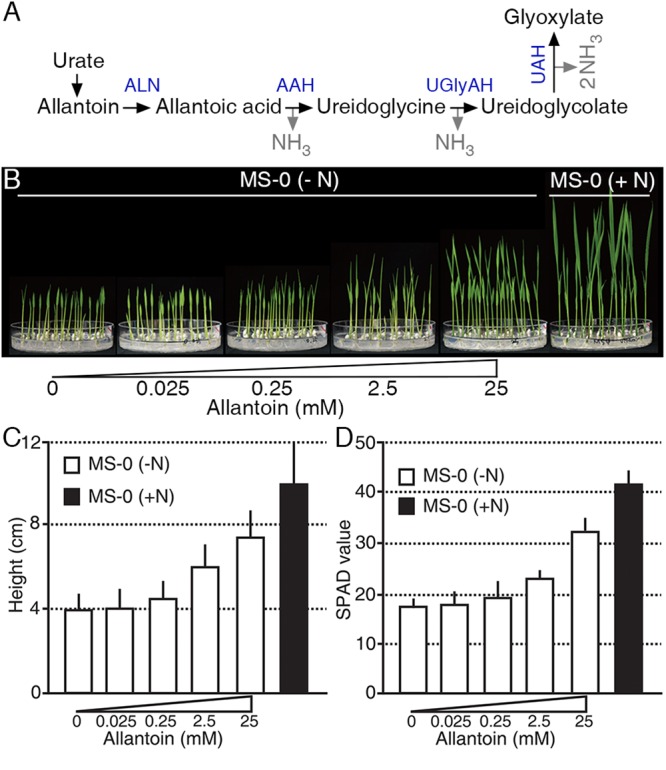
Allantoin is used as an N source in rice. **(A)** The allantoin catabolic pathway. A molecule of allantoin is converted to glyoxylate and produces four molecules of NH_3_. ALN, ALLANTOINASE; AAH, ALLANTOATE AMIDOHYDROLASE; UGlyAH, UREIDOGLYCINE AMINOHYDROLASE; UAH, UREIDOGLYCOLATE AMINOHYDROLASE. **(B)** Phenotype (9-day-old), **(C)** height (9-day-old), and **(D)** SPAD value (16-day-old) of rice plants grown on growth media with various allantoin concentrations as the sole N source. Values represent the mean + SD of two biological replicates. Each test used 20 plants for each allantoin concentration.

Ureides, such as allantoin and allantoic acid, are used as major N sources in legumes (Todd et al., 2006; Raso et al., 2007; Baral et al., 2016) (**Figure 1A**); however, little is known about the roles of ureides as an N source in non-legumes. To investigate an N source role of allantoin in rice, we treated plants with various concentrations of allantoin as the sole N source for 9 days (**Figure 1B**). Low concentrations of allantoin (0.025 and 0.25 mM) did not affect rice growth, which was similar to that of plants receiving no N supplement. However, rice plants treated with 2.5 mM allantoin showed enhanced growth and development compared with those exposed to lower concentrations (**Figures 1B,C**). Treatment with 25 mM allantoin resulted in a significant increase in seedling height (**Figure 1B**), such that the height was ∼75% of the height of plants grown on GM including 20 mM ammonium nitrate and 19 mM potassium nitrate (**Figure 1C**). To investigate whether allantoin treatment affects leaf N status, we monitored SPAD-based chlorophyll content in 16-day-old rice plants grown on GM including various concentrations of allantoin as the sole N source (**Figure 1D**). As with seedling height, low concentrations of allantoin did not affect chlorophyll content similar to no N supplied plants. However, over 2.5 mM allantoin treatment resulted in a substantial increase in chlorophyll levels, similar to those in rice plants grown on GM including 20 mM ammonium nitrate and 19 mM potassium nitrate (**Figure 1D**). These results indicate that the rice growth pattern and leaf N status positively correlate with allantoin levels.

### Levels of Allantoin Metabolites Rapidly Change in Response to the N Status

**FIGURE 2:**
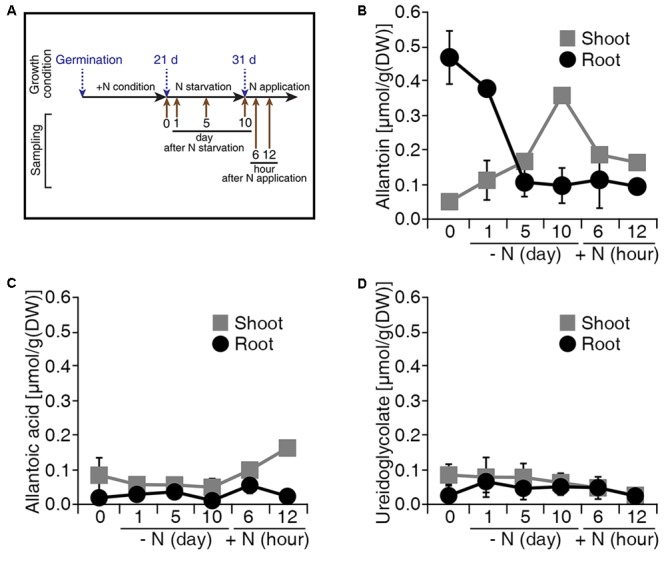
Levels of allantoin metabolites are sensitive to N status in rice. **(A)** Design of the N starvation and re-application experiment. Dongjin wild type plants were grown for 21 days on soil and hydroponic Yoshida solution with different N sources. Seedlings were transferred to N starvation conditions for 10 more days, which consisted of the Yoshida solution without N sources. After N starvation, NH_4_NO_3_ was added to the solution (2.8 mM final concentration), defined as the N re-application condition. Levels of allantoin **(B)**, allantoic acid **(C)**, and ureidoglycolate **(D)** in shoots and roots in response to N starvation and re-application. Values represent the mean + SE of two biological and two technical replicates.

Allantoin is converted to glyoxylate by four enzymatic reactions in a catabolic process that produces four molecules of ammonium, which represent a source of N (**Figure 1A**). To investigate the production of allantoin-derived metabolites in response to changes in exogenous N status, we measured the levels of allantoin-derived metabolites during N starvation and re-application conditions (**Figure 2A**). Under conditions with sufficient N, rice plants accumulated higher levels of allantoin metabolite in roots than in shoots (**Figure 2B**). When the plants were transferred to N starvation conditions, allantoin metabolite levels in the roots gradually decreased, but gradually increased in the shoots. At 5 days after N starvation, allantoin levels in the roots and shoots were ∼4.5-fold less and ∼3.5-fold more, respectively, than in the 0 h N starvation sample. Furthermore, allantoin metabolite levels in the shoots declined when N sources were re-applied to the plants (**Figure 2B**). Levels of allantoic acid, ureidoglycolate and glyoxylate remained unchanged during the N starvation and re-application (**Figures 2C,D** and Supplementary Figure S1). These data suggest that allantoin concentration may be a limiting factor in the generation of allantoin-derived metabolites in response to N status in rice plants.

### *OsALN* and *OsUPS1* Show Opposite Patterns of Expression in Response to N Status

**FIGURE 3:**
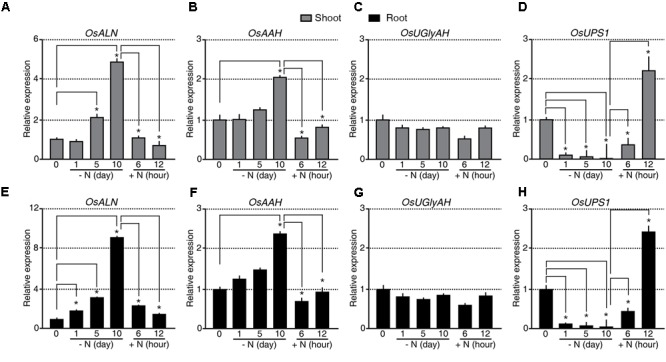
Expression of *OsALN* and *OsUPS1* changes rapidly in response to changing N status. qRT-PCR analysis of *OsALN* expression in shoots **(A–D)** and roots **(E–H)** following N starvation and re-application treatments **(A,E)**, *OsAAH* **(B,F)**, *OsUGlyAH* **(C,G)**, and *OsUPS1* **(D,H)**. *OsUBI1* expression was used as an internal control. Data shown are the mean + SD of two biological and two technical replicates. Asterisks indicate significant differences based on a 95% confidence interval by a Student’s *t*-test.

The rice genome is predicted to contain singles gene for each of the allantoin-catabolic enzymes: *ALLANTOINASE* (*OsALN*, Os04g0680400), *ALLANTOATE AMIDOHYDROLASE* (*OsAAH*, Os06g0665500), *UREIDOGLYCINE AMINOHYDROLASE* (*OsUGlyAH*, Os07g0495000), and *UREIDOGLYCOLATE AMIDOHYDROLASE* (*OsUAH*, Os12g0597500). To understand the transcriptional regulation of these allantoin-catabolic genes in response to the N status, qRT-PCR analysis was carried out using the tissues from the N starvation and re-application experiment (**Figure 2A**). A significant difference in expression was defined as a fold change in transcript levels of <-2.0 or >2.0 and *P*-value < 0.05. *OsALN* expression started to increase in shoots 5 days after N starvation and in roots soon after N starvation (**Figures 3A,E**). *OsALN* expression decreased rapidly in both shoots and roots after N re-application. *OsAAH* expression increased in both shoots and roots at 10 days after N starvation and decreased in both organs soon after N re-application (**Figures 3B,F**). In contrast, *OsUGlyAH* and *OsUAH* did not show any change in expression during N starvation and re-application (**Figures 3C,G** and Supplementary Figure S1). These data suggest that allantoin is rapidly catabolized under N starvation conditions, while levels are restored in rice organs by a down-regulation of allantoin catabolic genes under N re-application conditions.

However, an alternative explanation for the observed allantoin accumulation in the shoots and its reduction in the roots during N starvation is root-to-shoot remobilization of allantoin. To test this possibility, we investigated the transcriptional regulation of *UREIDE PERMEASE 1* (*OsUPS1*, Os12g0503000), a putative allantoin transporter, during N starvation and re-application (**Figures 3D,H**). Expression of *OsUPS1* decreased substantially in both shoots and roots soon after N starvation and was quickly up-regulated in both organs soon after N re-application (**Figures 3D,H**), suggesting that allantoin accumulation in shoots during N starvation was not caused by allantoin root-to-shoot remobilization by UPS1. We verified the expression patterns of *OsALN* and *OsUPS1* in response to the N status in an independent N starvation and re-application experiment (Supplementary Figure S2). Even the transcriptional regulation of *OsALN* and *OsUPS1* showed quick response dynamics as a nitrate transporter, *OsNRT* that is one of early response genes to different N status (Supplementary Figure S2). Taken together, the data indicate that the expression levels of *OsALN* and *OsUPS1* are negatively correlated with each other in response to exogenous N status.

### The Molecular N Sensors, *proUPS1::UPS1-LUC2* and *proALN::ALN-LUC2*

**FIGURE 4:**
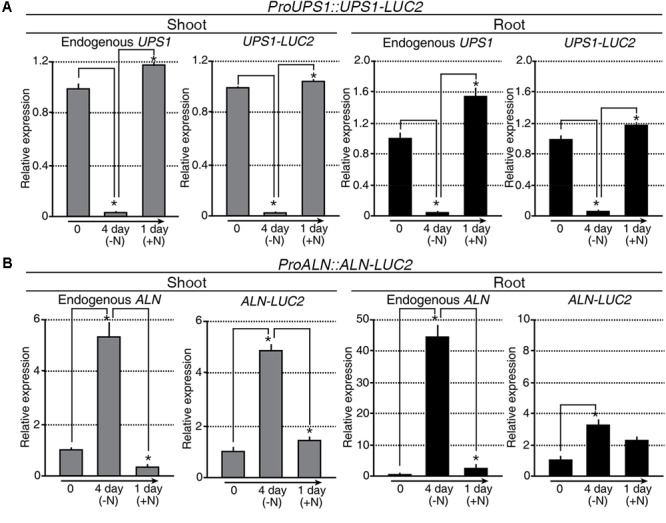
Transcriptional regulation of *proALN::ALN-LUC2* and *proUPS1::UPS1-LUC2* is similar to the expression of endogenous genes modulated by differences in N status. qRT-PCR analysis of *proUPS1::UPS1-LUC2* **(A)** and *proALN::ALN-LUC2* **(B)** expression in shoots and roots following N starvation and re-application treatments. *UPS1-LUC2* and *ALN-LUC2* correspond to transcripts derived from *proUPS1::UPS1-LUC2* and *proALN::ALN-LUC2*, respectively. *OsUBI1* expression was used as an internal control. Data shown are the mean + SD of two biological and two technical replicates. Asterisks indicate significant differences based on a 95% confidence interval by a Student’s *t*-test.

Taking advantage of *OsALN* and *OsUPS1* expression patterns, we generated the *in vivo* molecular N sensors *proALN::ALN-LUC2* and *proUPS1::UPS1-LUC2* to monitor N status in transgenic rice plants (Supplementary Figures S3A,B). Among 70 *proALN::ALN-LUC2* and 66 *proUPS1::UPS1-LUC2* T_0_ transgenic lines (Supplementary Figures S3C,D), we focused on16 *proALN::ALN-LUC2* and 18 *proUPS1::UPS1-LUC2* lines, which contained single copies of the transgenes, as confirmed by Taqman PCR (Supplementary Figures S3E–H). We then randomly selected five independent T_2_ homozygous lines for each molecular N sensor and used the T_3_ generation for further studies. qRT-PCR analysis was carried out to assess the transcriptional regulation of the molecular N sensors in response to the N status during N starvation and re-application experiments (**Figure 4**). Endogenous *UPS1* gene expression and *UPS1-LUC2* transgene expression in the *proUPS1::UPS1-LUC2* plants showed identical patterns in response to N status (**Figure 4A**). Specifically, both genes were down-regulated in the transgenic plants under N starvation, but were up-regulated after N re-application. In the *proALN::ALN-LUC2* plants, the endogenous *ALN* gene and the *ALN-LUC2* transgene also showed identical expression responses to the N status in the shoots (**Figure 4B**): they were up-regulated in the transgenic plants during N starvation, but down-regulated after N re-application. However, we observed different responses to N status in the roots of *proALN::ALN-LUC2* plants (**Figure 4B**). Endogenous *ALN* was substantially up-regulated (45-fold) in roots under N starvation, whereas the *ALN-LUC2* transgene was only slightly up-regulated (3-fold). From this we infer that the *proALN::ALN-LUC2* construct did not contain a regulatory region that controls gene expression in response to N levels in roots. We concluded that the transcriptional regulation of *proALN::ALN-LUC2* and *proUPS1::UPS1-LUC2* in the transgenic plants was sensitive to exogenous N status.

### Transgenic Plants Expressing the *proUPS1::UPS1-LUC2* Sensor Show Strong Luciferase Activity Under High N Conditions

**FIGURE 5:**
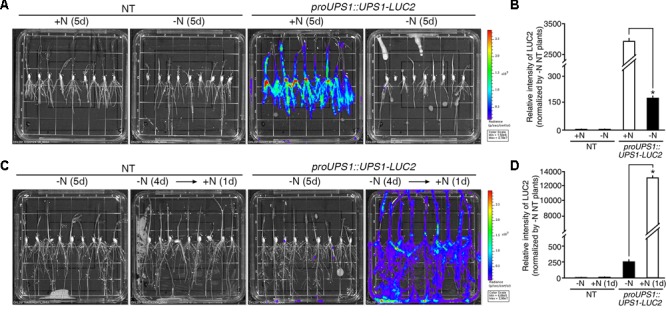
Luminescence resulting from *proUPS1::UPS1-LUC2* is strong under high N conditions. **(A)** Images of 5-day-old NT (non-transgenic) and *proUPS1::UPS1-LUC2* plants grown on GM+N (growth media +N) or GM–N. **(B)** Relative intensity of the luminescence in T_3_ homozygous *proUPS1::UPS1-LUC2* plants under the same conditions as in **(A)**. **(C)** Images of 5-day-old NT and *proUPS1::UPS1-LUC2* plants grown on GM–N for 5 days, or grown on GM–N for 4 days followed by addition of 100 mM ammonium nitrate for 1 day. **(D)** Relative intensity of the luminescence in T_3_ homozygous *proUPS1::UPS1-LUC2* plants under the same conditions as in **(C)**. Data shown are the mean + SD of 5 T_3_ homozygous *proUPS1::UPS1-LUC2* lines (*n* = 10 for each line). NT plants grown on GM–N were used as a control for normalization. Asterisks indicate significant differences based on a 95% confidence interval by a Student’s *t*-test.

Two methods were used to test the molecular N sensor abilities of the *proUPS1::UPS1-LUC2* plants: long-term incubation under high N conditions and short-term incubation under high N conditions. For long-term incubation, transgenic rice plants were grown on media with high levels of N sources, including 20 mM ammonium nitrate and 19 mM potassium nitrate (GM+N), or on growth media without N sources (GM-N) for 5 days (**Figure 5A**). High levels of luciferase activity were detected in the *proUPS1::UPS1-LUC2* plants grown on the GM+N compared with plants grown on the GM-N. To quantify the luminescence signals, we tested five independent homozygous lines (#11, 16, 33, 40, and 42), which had single copies of the transgene. Luciferase activity in the *proUPS1::UPS1-LUC2* plants grown on GM+N was ∼2,800-fold stronger than that in the NT controls and ∼20-fold stronger than that of the *proUPS1::UPS1-LUC2* plants grown on GM-N (**Figure 5B** and Supplementary Figure S4A). In the short-term incubation experiment, transgenic rice plants were grown on GM-N for 4 days then for one additional day in the same medium but including 100 mM ammonium nitrate (**Figure 5C**). As in the long-term incubation experiment, luciferase activity was higher in the *proUPS1::UPS1-LUC2* plants by 1 day after growth in the elevated N conditions than in plants grown on GM-N. We also quantified the luminescence in five independent homozygous lines. Luciferase activity in *proUPS1::UPS1-LUC2* plants 1 day after incubation in high N was ∼13,000-fold greater than in the NT controls and ∼50-fold greater than in the *proUPS1::UPS1-LUC2* plants grown on GM-N (**Figure 5D** and Supplementary Figure S4B). These data indicate that the *proUPS1::UPS1-LUC2* sensor detects high exogenous N status, resulting in increased luminescence signals.

### The Luminescent Signal of the *proALN::ALN-LUC2* Sensor Is Quickly Reduced Under High N Conditions

**FIGURE 6:**
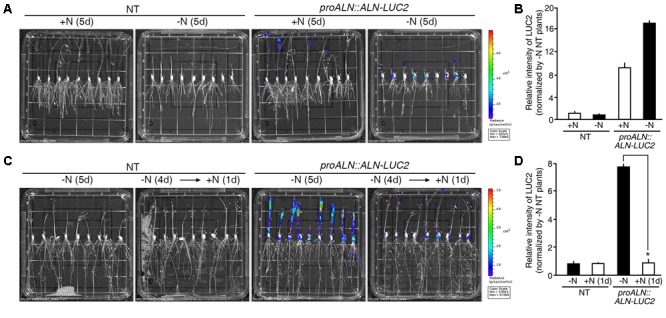
Luminescence resulting from *proALN::ALN-LUC2* plants is strong under low N conditions. **(A)** Images of 5-day-old NT (non-transgenic) and *proALN::ALN-LUC2* plants grown on GM+N (growth media +N) or GM–N media. **(B)** Relative intensity of the luminescence in T_3_ homozygous *proALN::ALN-LUC2* plants under the same conditions as in **(A)**. **(C)** Images of 5-day-old NT and *proALN::ALN-LUC2* plants grown on GM–N for 5 days, or grown on GM–N for 4 days followed by addition of 100 mM ammonium nitrate for 1 day. **(D)** Relative intensity of the luminescence in T_3_ homozygous *proALN::ALN-LUC2* plants under the same conditions as in **(C)**. Data shown are the mean + SD of 5 T_3_ homozygous *proALN::ALN-LUC2* lines (*n* = 10 for each line). NT plants grown on GM–N were used as a control for normalization. Asterisks indicate significant differences based on a 95% confidence interval by a Student’s *t*-test.

During long-term incubation experiment, luciferase activity was detected in *proALN::ALN-LUC2* plants grown on the GM+N or GM-N, but we observed no difference in activity between plants grown on the GM+N and the GM-N media (**Figure 6A**). To quantify the luminescence signals, we tested five independent homozygous lines (#5, 11, 16, 30, and 51), which had single copies of the transgene. Luciferase activity in the *proALN::ALN-LUC2* plants grown on GM-N was ∼17-fold greater than in the NT controls and ∼1.8-fold greater than in the *proALN::ALN-LUC2* plants grown on GM+N (**Figure 6B** and Supplementary Figure S4C). It was therefore difficult to distinguish between the effects of growth on GM+N and GM-N. However, under short-term incubation, luciferase activity was higher in *proALN::ALN-LUC2* plants continuously grown on GM-N than in those incubated for 1 day with high N (**Figure 6C**). We quantified the luminescence of five independent homozygous lines. Luciferase activity in *proALN::ALN-LUC2* plants continuously grown on GM-N was ∼8-fold greater than that in the NT controls and ∼7-fold stronger than in *proALN::ALN-LUC2* plants grown for 1 day with high N (**Figure 6D** and Supplementary Figure S4D). These results indicate that the *proALN::ALN-LUC2* sensor detects the early response to high N conditions, resulting in a rapid reduction in the luminescence signal.

### The *proUPS1::UPS1-LUC2* and *proALN::ALN-LUC2* Sensors Respond Non-selectively to Different N Sources

**FIGURE 7:**
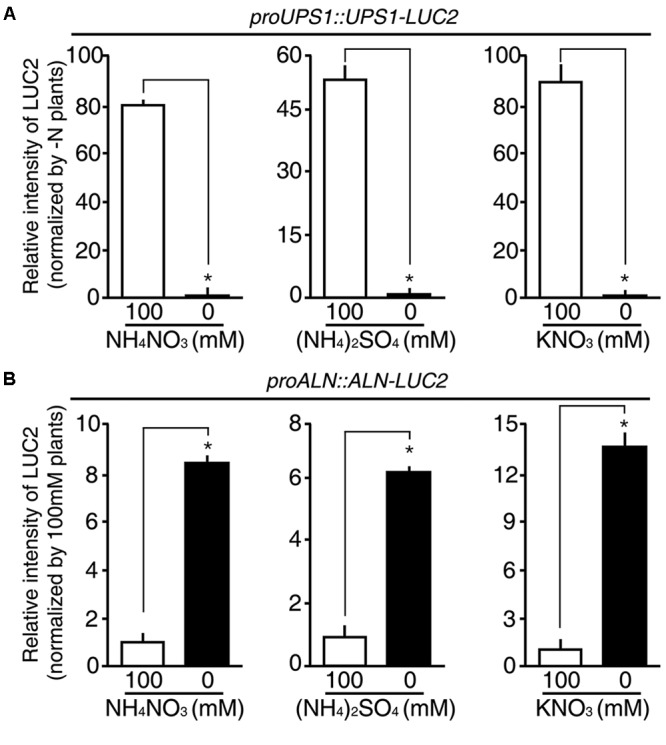
N substrate specificity of *proUPS1::UPS1-LUC2* and *proALN::ALN-LUC2* in rice. Relative intensity of the luminescence in T_3_ homozygous *proUPS1::UPS1-LUC2* **(A)** and *proALN::ALN-LUC2* **(B)** plants grown on GM–N (growth media –N) for 4 days, followed by addition of 100 mM ammonium nitrate, ammonium sulfate or potassium nitrate for 1 day. Data shown are the mean + SD of 5 T_3_ homozygous lines for each N sensor (*n* = 10 for each line).

Since rice plants take up both ammonium and nitrate through their roots, we hypothesized that the *proUPS1::UPS1-LUC2* and *proALN::ALN-LUC2* plants might show specific responses to these different sources of N. To address this, we treated 5-day-old *proUPS1::UPS1-LUC2* and *proALN::ALN-LUC2* plants grown on GM-N with 100 mM ammonium sulfate or with100 mM potassium nitrate as the sole N source, grew them for 1 more day, and measured luminescence. A 100 mM ammonium nitrate treatment was used as a positive control. As when treated with 100 mM ammonium nitrate, *proUPS1::UPS1-LUC2* plants showed strong luciferase activity after treatment with 100 mM ammonium sulfate, resulting in ∼50-fold higher activity than the mock treatments, while a 100 mM potassium nitrate treatment resulted in ∼90-fold higher activity than mock treatments (**Figure 7A**). In contrast, *proALN::ALN-LUC2* plants showed reduced luciferase activities when treated with 100 mM ammonium nitrate, resulting in ∼8.5-fold less activity than mock treated plants, ∼6-fold less when treated with 100 mM ammonium sulfate and ∼14-fold less when treated with 100 mM potassium nitrate (**Figure 7B**). These results indicate that *proUPS1::UPS1-LUC2* and *proALN::ALN-LUC2* respond non-selectively to N source levels, and can be used broadly in rice plants as molecular N sensors, able to detect N status modulated by a range of N sources.

### N Sensitivity of the *proUPS1::UPS1-LUC2* and *proALN::ALN-LUC2* Sensors

**FIGURE 8:**
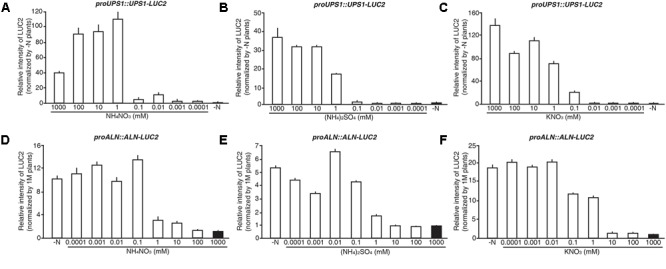
N responsive range of *proUPS1::UPS1-LUC2* and *proALN::ALN-LUC2* in rice plants. Relative intensity of the luminescent signals in T_3_ homozygous *proUPS1::UPS1-LUC2* **(A–C)** and *proALN::ALN-LUC2* **(D–F)** plants, which were grown on GM–N (growth media –N) for 4 days followed by the growth in 0, 0.0001, 0.001, 0.01, 0.1, 1, 10, 100, or 1,000 mM ammonium nitrate **(A,D)**, ammonium sulfate **(B,E)** and potassium nitrate **(C,F)** for 1 day. Data shown are the mean + SD of 3 T_3_ homozygous lines for each N sensor (*n* = 9 for each line).

To investigate the N sensitivity of the molecular N sensors, we treated 5-day-old *proUPS1::UPS1-LUC2* and *proALN::ALN-LUC2* plants grown on GM-N with various concentrations (1 M to 0.1 μM) of ammonium nitrate, ammonium sulfate or potassium nitrate as the sole nitrogen source for 1 additional day, before measuring their luminescence. *proUPS1::UPS1-LUC2* plants showed strong luciferase activities at ammonium nitrate, ammonium sulfate or potassium nitrate concentrations > 1 mM, whereas low nitrogen concentrations (<0.01 mM) resulted in weak luciferase activities (**Figures 8A–C**). Interestingly, 0.1 mM potassium nitrate induced strong luciferase activities in *proUPS1::UPS1-LUC2* plants, while 0.1 mM ammonium nitrate and ammonium sulfate did not. This suggests that the *proUPS1::UPS1-LUC2* sensor monitors high N status, indicated by strong luminescent activity, and low N status, as indicated by weak luminescent activity.

*proALN::ALN-LUC2* plants showed strong luciferase activities under low N concentrations (<0.1 mM), whereas *proALN::ALN-LUC2* plants showed weak activity under high N concentrations (>10 mM) (**Figures 8D–F**). Additionally, 1 mM ammonium nitrate and ammonium sulfate induced weak luciferase activity in *proALN::ALN-LUC2* plants, while 1 mM potassium nitrate induced strong activity. This suggests that the *proALN::ALN-LUC2* sensor can reflect high N status in rice plants, indicated by weak luminescent activity, and low N status, indicated by strong luminescent activity. These observations indicate that *proUPS1::UPS1-LUC2* and *proALN::ALN-LUC2* are effective N molecular sensors in rice plants responded by exogenous N status.

### Luminescence Signals of the Molecular Sensors Reflect Internal N Status in Rice

**FIGURE 9:**
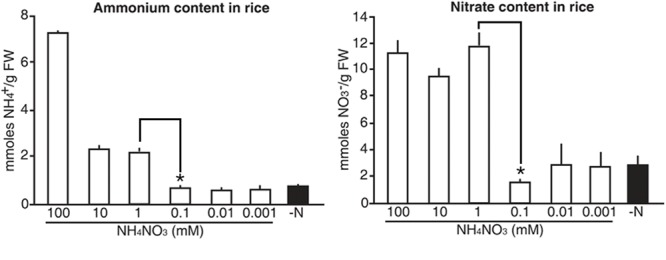
Internal levels of ammonium and nitrate that vary upon exogenous application of N sources in rice. Levels of ammonium and nitrate in wild type rice plants grown on GM–N (growth media –N) for 4 days followed by +N at 0, 0.001, 0.01, 0.1, 1, 10, or 100 mM ammonium nitrate for 1 day. Data shown are the mean + SD of two biological and three technical replicates. Asterisks indicate significant differences based on a 95% confidence interval by a Student’s *t*-test.

Finally, we tested whether the luminescence signals from the molecular sensors reflected internal N status in rice. Five-day-old wild type rice plants were grown on GM-N with various concentrations (100 mM to 1 μM) of ammonium nitrate as the sole nitrogen source for 1 additional day, before measuring internal levels of ammonium and nitrate. Exogenous application of high N sources (>1 mM) increased internal levels of ammonium and nitrate in rice plants by three and fourfold greater than in plants applied with low N sources (<0.1 mM), respectively (**Figure 9**). However, internal levels of ammonium and nitrate in rice plants remained unchanged upon exogenous application of low N sources (<0.1 mM). These data are consistent with luminescence signals of the N molecular sensors, in that the *proUPS1::UPS1-LUC2* and the *proALN::ALN-LUC2* showed strong luminescence under high N conditions (>1 mM) and low N conditions (<0.1 mM), respectively (**Figure 8**). Taken together, these results indicate that luminescence signals of the N molecular sensors reflect internal N status in rice.

## Discussion

### Allantoin Is Used as an N Source in Rice Plants

Ureides comprise up to 90% of the total N source in legumes (Herridge et al., 1978). However, it is not known whether allantoin also serves as an N source in non-legumes. Although allantoin has been reported to provide an N source in *A. thaliana* (Desimone et al., 2002; Yang and Han, 2004), this is still debatable. In this study, we investigated the role of allantoin in rice, using it as the sole N source. We observed that indeed rice utilized allantoin as an N source, and plants treated with 25 mM allantoin grew to ∼75% of the height and showed ∼79% of chlorophyll number of rice plants grown on 20 mM ammonium nitrate and 19 mM potassium as plants grown under ample N conditions. Given the proportional relationship between chlorophyll levels and leaf N content (Evans, 1983), these data indicated that allantoin can provide a source of N in rice. In addition, we detected allantoin-derived metabolites following the up-regulation of *OsALN* and *OsAAH* expression, under N starvation conditions. Allantoin catabolism generates ammonium, which is consistent with allantoin being used as an N source.

### Modulation of Ureide Metabolites and Allantoin Catabolic Genes During N Starvation

We observed that levels of allantoin were substantially reduced in rice roots under N starvation conditions, which correlated with the upregulation of *OsALN* expression. In contrast, allantoin concentration gradually increased in rice shoots under N starvation conditions. Since expression of *OsALN* was also induced in shoots at 5 days after N starvation, we conclude that allantoin must also be catabolized to generate ammonium in shoots. However, allantoin accumulation in shoots cannot be explained by allantoin catabolism, and one explanation is that allantoin may be synthesized in shoots under N starvation conditions. It was reported that under stress conditions, such as drought and high salinity, allantoin accumulates to high levels in *A. thaliana* leaves due to the activation of allantoin biosynthetic genes and/or repression of *ALN* expression (Irani and Todd, 2016). Since we found that *OsALN* was upregulated under N starvation conditions, it is possible that N starvation activates expression of allantoin biosynthetic genes to elevate the levels of the allantoin in the shoots. Another possibility is that root-to-shoot transport of allantoin is activated under N starvation conditions, and to test this possibility, we measured the expression of *OsUPS1*, an allantoin transporter. Expression of *OsUPS1* was rapidly down-regulated in both roots and shoots soon after N starvation, indicating that allantoin accumulation in shoots during N starvation is not caused by OsUPS1-mediated allantoin transport. Nevertheless, it is still possible that allantoin accumulation in shoots during N starvation may be caused by root-to-shoot mobilization of allantoin via UPS proteins, because rice genome has 4 *UPS* genes including *OsUPS1*. We therefore conclude that the pattern of allantoin accumulation can be explained by the altered expression of *OsUPS1* and *OsALN* in response to the N status. Under high N conditions, allantoin is mobilized to its target sites by activation of OsUPS1-mediated transport, coincident with down-regulation of OsALN-mediated catabolism. In contrast, under low N conditions allantoin is utilized locally as an N source via the OsALN-mediated catabolism, while OsUPS1-mediated transport is repressed.

### Molecular N Sensor for Monitoring the Internal N Status in Rice Plants

Lack of an N-specific phenotype is one important reason why N-related research is slow. This is because N phenotype can vary depending on exogenous levels of N sources (Han et al., 2015). In addition, detection of internal N status in plants is considered to be a major challenge. In this study, we developed a molecular N sensor system in rice to monitor internal N status in rice. We used the firefly *LUCIFERASE* reporter gene, which can be used to generate bioluminescence in living plants, and has been used to study specific environmental and hormonal responses (Alvarado et al., 2004). We took advantage of the responses of *OsALN* and *OsUPS1* to N status, and generated the *in vivo* molecular N sensors, *proALN::ALN-LUC2* and *proUPS1::UPS1-LUC2* in transgenic rice plants. These sensors showed sensitive yet non-selective responsiveness to N sources, in the form of ammonium and nitrate. Transgenic rice plants expressing *proUPS1::UPS1-LUC2* showed strong luminescence under high exogenous N conditions (>1 mM), whereas transgenic rice plants expressing the *proALN::ALN-LUC2* showed strong luminescence under low exogenous N conditions (<0.1 mM). Importantly, the range of the response is similar to that of physiological and developmental response of rice shown by exogenous N application (Feng et al., 2011; Fang et al., 2013; Hu et al., 2015; Li et al., 2017; Wang et al., 2018). The application with high N conditions (>1 mM) increased internal levels of ammonium and nitrate in rice plants by over threefold. Interestingly, exogenous levels of N application at between 1 mM and 100 μM are the threshold to make the internal levels of N sources changed. The molecular N sensors can sensitively detect the threshold and display different luminescence signals. Thus, luminescence signals of the molecular N sensors reflect internal N status in rice. We conclude that *proALN::ALN-LUC2* and *proUPS1::UPS1-LUC2* can be utilized as sensors for various fields of N research to monitor internal N status in rice.

## Author Contributions

D-KL and J-KK conceived the project, designed the experiments, supervised the project, and wrote the manuscript. D-KL carried out the experiments with assistance from MR, HJ, SC, and YSK. All authors analyzed and discussed the results.

## Conflict of Interest Statement

The authors declare that the research was conducted in the absence of any commercial or financial relationships that could be construed as a potential conflict of interest.
