# Supplementary material for: A Nitrogen Molecular Sensing System, Comprised of the ALLANTOINASE and UREIDE PERMEASE 1 Genes, Can Be Used to Monitor N Status in Rice
Source: Front Plant Sci. 2018 Apr 18;9:444. doi: 10.3389/fpls.2018.00444 (PMC5915567; doi:10.3389/fpls.2018.00444)
Supplement: Supplementary file 1 [file Image_1.PDF]

# Supplementary Material

# A nitrogen molecular sensing system, comprised of the *ALLANTOINASE* and *UREIDE PERMEASE 1* genes, can be used to monitor N status in rice

Dong-Keun Lee*, Mark Christian Felipe R. Redillas, Harin Jung, Seowon Choi, Youn Shic Kim, and Ju-Kon Kim*

*** Correspondence:** Corresponding Author: jukon@snu.ac.kr and eastrootut@snu.ac.kr

**Supplementary Table S1.** List of gene specific primers for qRT-PCR

| Target gene (ID) | Forward primers | Reverse primers |
|---|---|---|
| *OsALN* (Os04g0680400) | GGACTACGGTGACGCGGTTA | CAGCTGCTGCTCTTGTACCA |
| *OsAAH* (Os06g0665500) | CAACTTTTCTGGGAAGCGCC | GAGCATTAGCAGTGCCCTCA |
| *OsUGlyAH* (Os07g0495000) | CAGCCCTACCACCAAAAGGT | GGCATAAGAGTCCACCAGCA |
| *OsUAH* (Os12g0597500) | ACGTGCAAGCTCGAAGGTAT | AGTTGCAACCGCTCCTAGTC |
| *OsUPS1* (Os12g0503000) | GCCTGCCTTGGATCTCTTGT | GGAGGTGCTTGGTGAGTTCT |
| *LUC2* | TACCGCTTCGAGGAGGAGCTATTCT | ATCTCGTGCAAGTTGCTTAGGTCGT |
| *OsNRT* (Os01g0704100) | GACGGAGACCGGGATCAAGTACAT GGG | CCACTCCCGGCTGTAGTACTCCTCC |
| *OsUBI1* (Os06g0681400) | ATGGAGCTGCTGCTGTTCTA | TTCTTCCATGCTGCTCTACC |

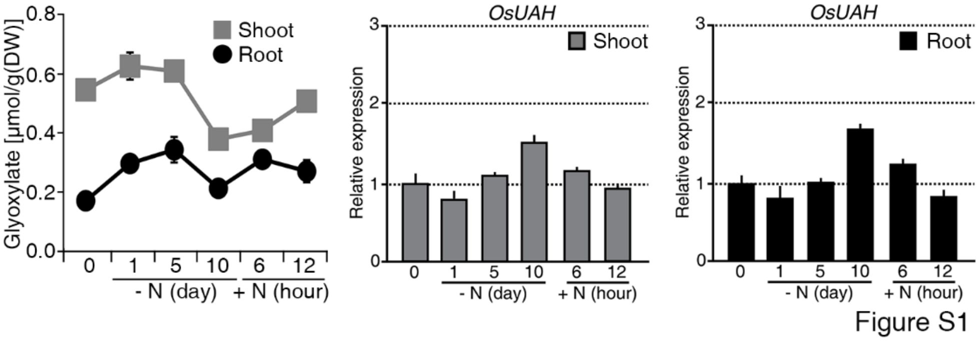


**Supplementary Figure S1.** Level of glyoxylate and *OsUAH* expression during N starvation and re-application during the experiments shown in Figure 2A. Values represent the mean + SE of two biological and two technical replicates.

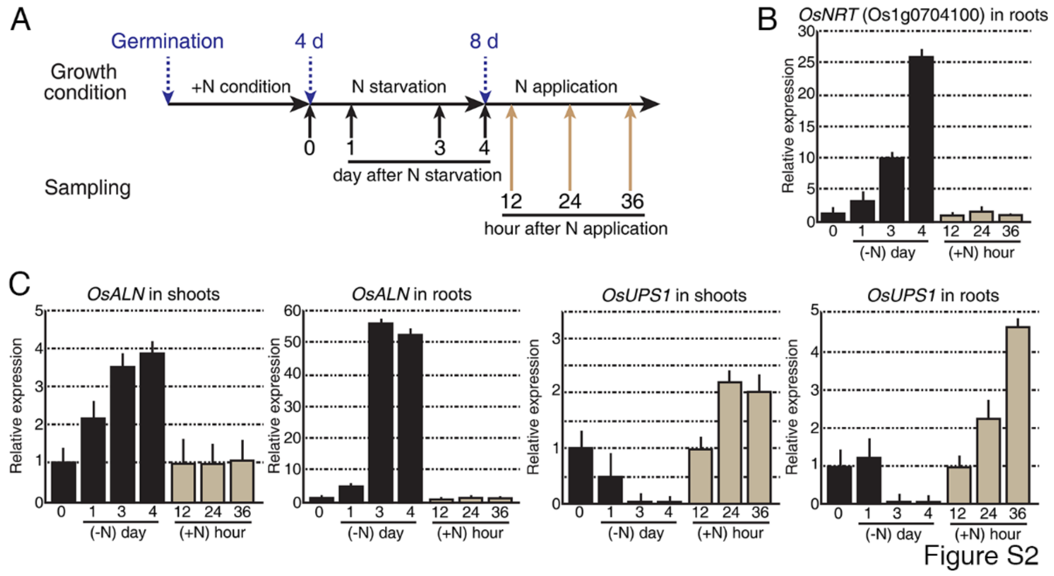


**Supplementary Figure S2.** Expression levels of *OsALN* and *OsUPS1* change rapidly in response to different N concentrations. **(A)** Design of the N starvation and re-application experiment. Dongjin rice plants were germinated and grown on wet paper with MS salts (+N) for 4 days. For the N starvation treatment, Dongjin rice plants were transferred to GM-N and grown for 4 days. For the N re-application treatment, plants were grown in 100 mM ammonium nitrate for additional 2 days. Shoots and roots were harvested at 0, 1, 3 and 4 days during N starvation and at 12, 24 and 36 h during N re-application. Expression levels of *OsNRT* (Os1g0704100) **(B)**, *OsALN* and *OsUPS1* **(C)** in shoots and roots harvested following the N starvation and re-application treatments. *OsUBI1* expression was used as an internal control. Values represent the mean + SD of three biological and two technical replicates.

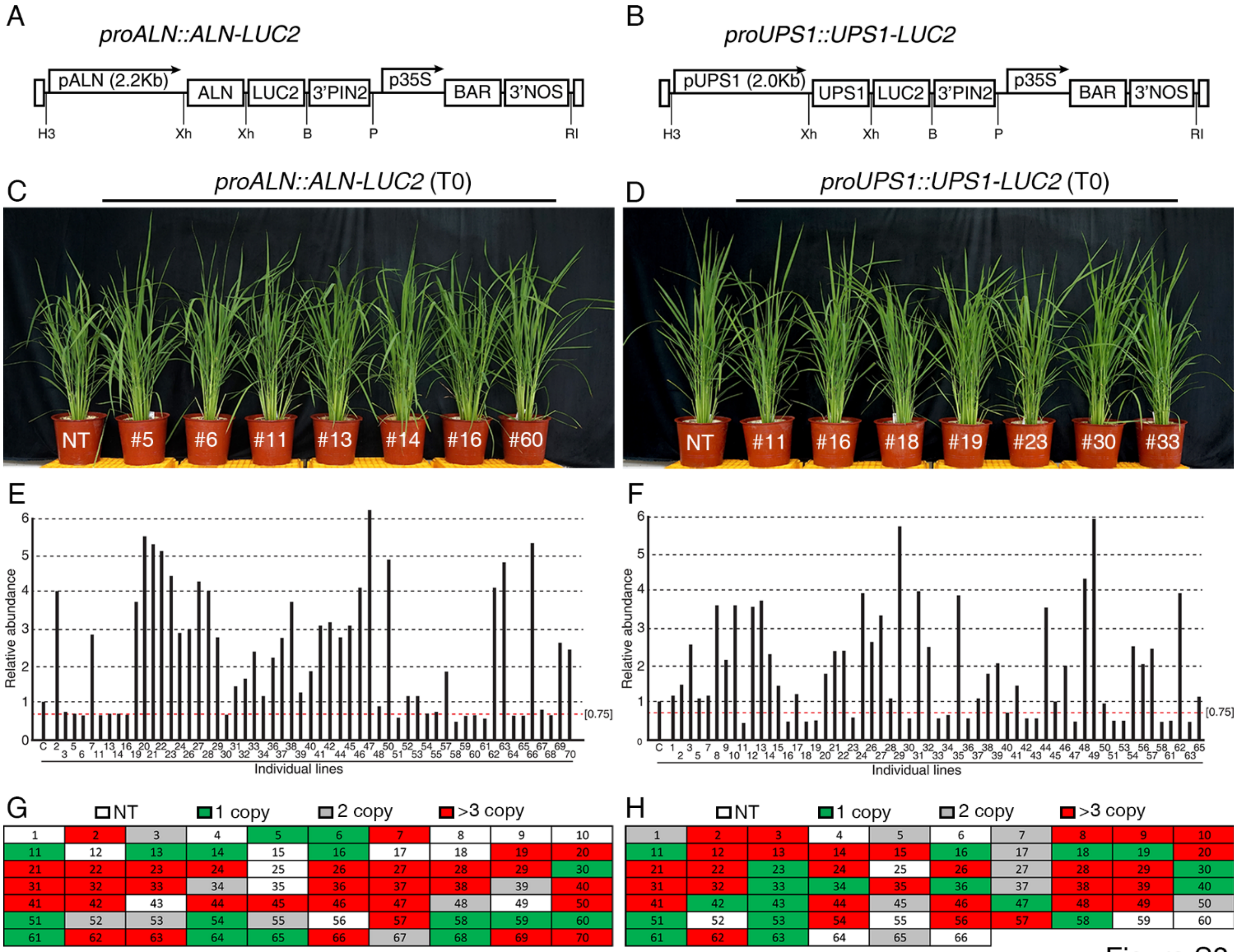


Figure S3

**Supplementary Figure S3.** Generation of *proALN::ALN-LUC2* and *proUPS1::UPS1-LUC2* transgenic rice plants. Vector maps for *proALN::ALN-LUC2* **(A)** and *proUPS1::UPS1-LUC2* **(B)**. Phenotypes of T0 *proALN::ALN-LUC2* **(C)** and *proUPS1::UPS1-LUC2* **(D)** rice plants. TaqMan q-PCR analysis to detect the presence of the *NOS* terminator of $T_0$ *proALN::ALN-LUC2* **(E)** and *proUPS1::UPS1-LUC2* **(F)** rice plants. A oligonucleotide probe targeted to rice *TUBULIN 1* (*OsTub1*, Os11g0247300) was used as an internal control for q-PCR normalization. A transgenic line with a single-copy homozygous T-DNA insertion was used as a positive control (C). Summary of copy number of transgene in $T_0$ *proALN::ALN-LUC2* **(G)** and *proUPS1::UPS1-LUC2* **(H)** rice plants.

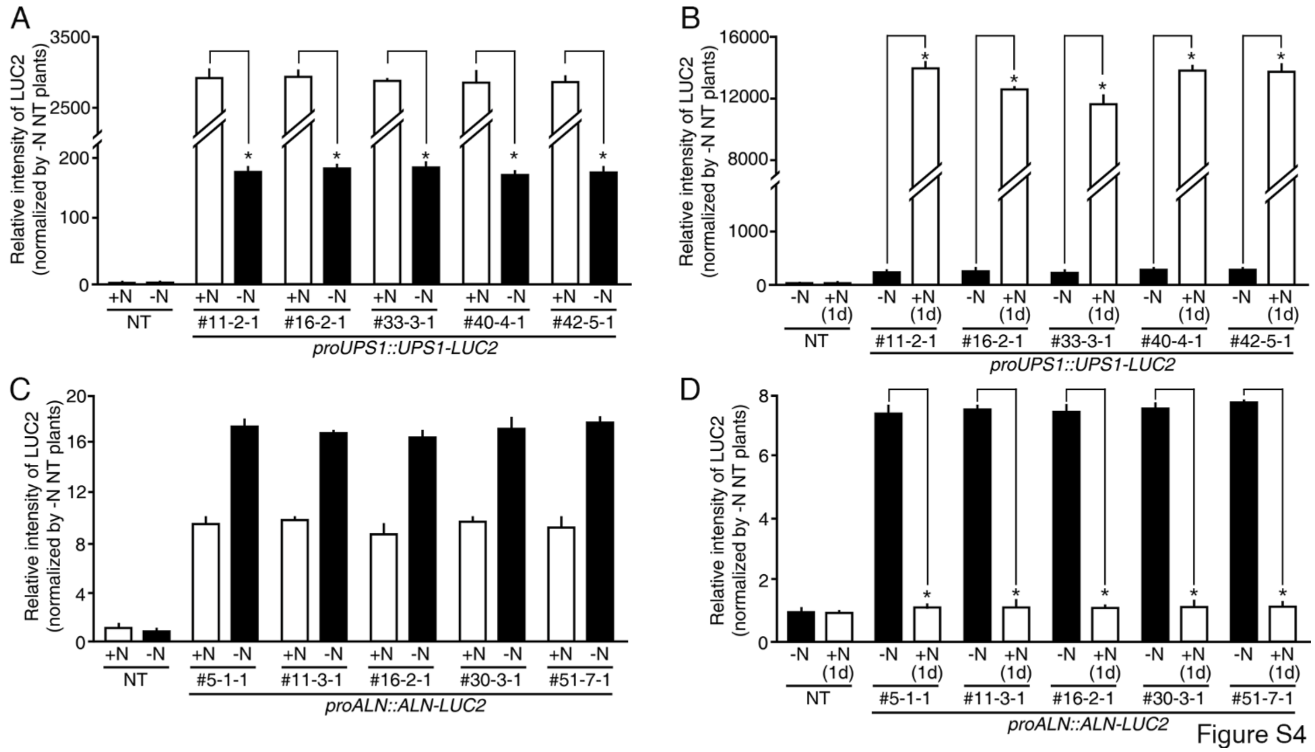


**Supplementary Figure S4.** Luminescence intensity of *proUPS1::UPS1-LUC2* and *proALN::ALN-LUC2* plants. Relative intensity of the luminescence in $T_3$ homozygous *proUPS1::UPS1-LUC2* **(A)** and *proALN::ALN-LUC2* **(C)** plants grown on the GM+N or GM-N for 5 days. Relative intensity of the luminescence in $T_3$ homozygous *proUPS1::UPS1-LUC2* **(B)** and *proALN::ALN-LUC2* **(D)** plants grown on the GM-N for 5 days or grown on the growth media with GM-N for 4 days, followed by growth in medium containing 100 mM ammonium nitrate for 1 day. Data are shown as the mean + SD (n= 10 plants). NT grown on the GM-N was used as a control for normalization. Asterisks indicate significant differences based on a 95 % confidence interval by a Student's t-test.
